# Supplementary material for: Excellent Temperature-Control Based on Reversible Thermochromic Materials for Light-Driven Phase Change Materials System
Source: Molecules. 2019 Apr 24;24(8):1623. doi: 10.3390/molecules24081623 (PMC6526475; doi:10.3390/molecules24081623)
Supplement: Supplementary File 1 [file molecules-24-01623-s001.pdf]

Table S1: Enthalpy of fusion of 1-HD, 1-tetradecanol and 1-dodecanol

	ΔH_m(J/g)
1-HD	233.8J/g
1-tetradecanol	210.1J/g
1-dodecanol	186J/g
